# Comparison of cell type distribution between single-cell and single-nucleus RNA sequencing: enrichment of adherent cell types in single-nucleus RNA sequencing

**DOI:** 10.1038/s12276-022-00892-z

**Published:** 2022-12-02

**Authors:** Jin-Mi Oh, Minae An, Dae-Soon Son, Jinhyuk Choi, Yong Beom Cho, Chang Eun Yoo, Woong-Yang Park

**Affiliations:** 1 Samsung Genome Institute, Samsung Medical Center, Seoul, Korea; 2 Innovative Institute for Precision Medicine, Samsung Medical Center, Seoul, Korea; 3 School of Big Data Science, Data Science Convergence Research Center, Hallym University, Chuncheon, 24252 Korea; 4 Department of Legal Medicine, College of Medicine, Korea University, Seoul, Korea; 5 Department of Surgery, Sungkyunkwan University, Samsung Medical Center, Seoul, Korea; 6 Department of Health Sciences and Technology, SAIHST, Sungkyunkwan University, Seoul, Korea; 7 Basic Research Support Center, Samsung Research Institute for Future Medicine/Samsung Medical Center, Seoul, Korea; 8 Geninus Inc, Seoul, Korea; 9 Department of Molecular Cell Biology, Sungkyunkwan University School of Medicine, Suwon, Korea

**Keywords:** Bioinformatics, Reverse transcription polymerase chain reaction

## Abstract

Single-cell ribonucleic acid (RNA) sequencing (scRNA-seq) is an effective technique for estimating the cellular composition and transcriptional profiles of individual cells from fresh tissue. Single-nucleus RNA sequencing (snRNA-seq) is necessary to perform this type of analysis in frozen or difficult-to-dissociate tissues, which cannot be subjected to scRNA-seq. This difference in the state of tissues leads to variation in cell-type distributions among each platform. To identify the characteristics of these methods and their differences, scRNA-seq and snRNA-seq were performed in parallel for colon and liver tissues. The two platforms revealed similar diversity but different proportions of cell types in matched tissues. The proportions of epithelial cells in the colon and hepatocytes in the liver were relatively high in snRNA-seq and that of immune cells was relatively high in scRNA-seq. This difference could be explained by variations in the expression scores of adhesion genes due to the disruption of the cytoplasmic contents during scRNA-seq. The enrichment of epithelial cells in the colon resulted in a discrepancy in the differentiation of epithelial cells. This enrichment was also well matched with the images of hematoxylin and eosin staining and the estimated distribution of cell types in bulk RNA sequencing. These results showed that snRNA-seq could be used to analyze tissues that cannot be subjected to scRNA-seq and provides more information in specific cell type analysis.

## RNA sequencing: Different methods skew toward different cell types

Methods that analyze single-cell RNA sequences in fresh clinical samples are skewed toward examining a different subset of cell types than methods analyzing single-nucleus sequences in frozen tissues. A team led by Chang Eun Yoo and Woong-Yang Park from the Samsung Medical Center in Seoul, South Korea, applied two different RNA-sequencing techniques in parallel on a common set of colon and liver tissues collected from cancer patients. They showed that single-cell profiling, which works best with fresh samples that can be readily dissociated into component cells, returned results on more immune cells, whereas single-nucleus methods, best for archived -frozen tissues, were enriched for more cells of epithelial origin. The different cell distributions revealed by this matched tissue analysis should help inform data interpretation and assay selection for future RNA-sequencing studies.

## Introduction

Due to recent advances in high-throughput droplet microfluidics technologies that can analyze thousands of individual cells in parallel, single-cell ribonucleic acid (RNA) sequencing (scRNA-seq) has become a powerful technology that can analyze gene expression in individual cells and reveal cell types, states, genetic diversity, and interactions in complex tumor ecosystems^1–6^. By using scRNA-seq to study organism development^7,8^, normal tissues^9,10^, cancer^4,5^, and other diseases^11,12^, researchers have gained deep insights into tissue heterogeneity and functions that were previously unavailable.

There are certain factors that must be considered in the scRNA-seq of clinical tissues^13^. First, immediate access to fresh tissue from surgical samples and rapid dissociation of these fresh tissues are necessary. Dissociation involves enzymatic digestion, which requires incubation. This leads to the alteration of gene expression by the cell transcriptional machinery and other environmental stresses^14,15^. Therefore, these artifacts and biases due to dissociation need to be considered when designing and analyzing data from single-cell experiments^16^. Second, this method cannot be applied to specific tissues that are difficult to dissociate or archived snap-frozen tissues for long-term storage. For example, it is difficult to dissociate individual cells from brain materials, and only frozen materials are available^17,18^.

To minimize dissociation-related artifacts and apply them to frozen tissue, snRNA-seq, which generates transcriptomic information from isolated nuclei, has been suggested as an alternative method^17–19^. Because much harsher conditions are adapted to release nuclei from tissue, snRNA-seq can be applied to frozen tissues as well as fresh tissues that cannot be successfully dissociated due to cell size and fragility^17,18^. It also enables multiplexed analyses of longitudinally archived samples from the same individual^20,21^. However, the amount of mRNA in nuclei is lower than that in cells, and it is challenging to enrich or deplete nuclei released from specific cell types of interest^13^. These factors may result in differences in the diversity and proportion of cell types identified in the tissue from the same individual between scRNA-seq and snRNA-seq^13,16,21,22^. Therefore, it is necessary to identify the reason for these differences between the two platforms to choose a suitable platform for research on the target tissue.

**Fig. 1:**
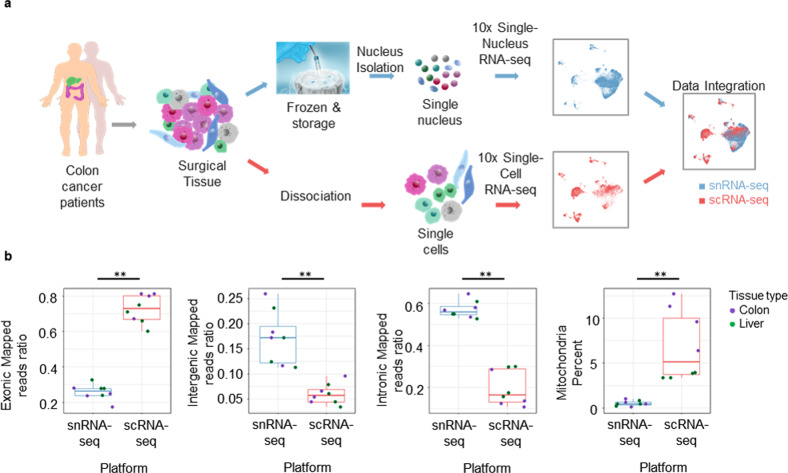
Experimental overview and comparison of each platform. **a** Schematic of the experimental design and analysis. Surgically resected tissues are split and processed in parallel for scRNA-seq and snRNA-seq. After the clustering of cell types based on each platform, the cell types of each cluster are integrated and compared. **b** Read ratios mapped to exonic, intergenic, and intronic regions according to the platform and the average percentage of mitochondrial reads per cell across platforms (**p ≤ 0.01; Wilcoxon signed-rank test).

To identify the characteristics of and differences between the two platforms, we performed scRNA-seq and snRNA-seq in parallel on colon and liver tissues (Fig. 1a). Comparison of the mapped read ratio and mitochondrial percentage showed typical results, in accord with the representative reports previously reported for each platform^13,21^ (Fig. 1b). Cell type profiling showed similarities in diversity but differences in proportion between each platform. The major difference in the proportion of cell types could be explained by the module scores of cell adhesion-related genes. The enrichment of epithelial cells in colon tissue by snRNA-seq could allow the discrepancies in the differentiation state of epithelial cells to be analyzed. Finally, pathological analysis and bulk sequencing by snRNA-seq were more accurate in terms of the proportion of cell types.

## Materials and methods

### Patient recruitment and samples

This study was reviewed and approved by the Institutional Review Board (IRB) of the Samsung Medical Center (SMC). All patients provided signed informed consent for the collection of specimens and detailed analyses of the derived genetic materials (Institutional Review Board no. 2017-07-131-017).

A total of eight tissue samples from five colorectal cancer patients, including two patients with liver metastasis, were obtained: SMC-22T, SMC-50N, SMC-50T, SMC-37LMN, SMC-37LMT, SMC-046N, SMC-046LMN, and SMC-099LMN (Supplementary Table 1).

### Single-cell and single-nucleus preparation for 10× sequencing

For scRNA-seq, single-cell suspensions of fresh colon and liver tissues were isolated by mechanical dissociation and enzymatic digestion within 16 h after surgery. Dissociation was performed using a tumor dissociation kit (Miltenyi Biotech, Germany) according to the manufacturer’s instructions. Briefly, tissues were cut into pieces of 2–4 mm in size and transferred to a C tube containing the enzyme mix (enzymes H, R, and A in RPMI 1640 medium). GentleMACS tissue dissociator programs h_tumor_01, h_tumor_02, and h_tumor_02 were run on a MACSmix tube rotator during two 30 min incubations at 37 °C. The resulting cell suspension was filtered through a 70 μm strainer and washed with RPMI 1640 medium, followed by Ficoll-Paque PLUS (GE Healthcare) separation. Each cell suspension was processed with 10× Chromium Single Cell 3′ Reagent Kits v3 (10× Genomics) according to the manufacturer’s protocol.

For snRNA-seq, tissue specimens were obtained from surgical excision without prior treatment and frozen in liquid nitrogen or at −80 °C for several months prior to nuclear preparation. Nuclear isolation buffers were prepared using standard nuclear isolation buffers (Nucleus EZ buffer, NUC-101, Sigma-Aldrich) and nuclear wash buffer supplemented with an RNase inhibitor (Promega). The prepared buffers were then placed on ice. Frozen tissues were cut with scissors into 1–2 mm pieces and transferred into a GentleMACS C tube containing 2 ml of ice-cold 1× lysis buffer. Dissociation was performed using the GentleMACS system, according to the m_Lung_01 and m_Lung_02 programs, after all the small pieces settled at the bottom of the tube on ice. The sample was briefly centrifuged for approximately 30 s and transferred to a 70 μm MACS SmartStrainer on a conical tube with a wide bore tip. After rinsing the filter with 4 ml of chilled wash buffer, the sample was mixed by inversion, and viability and the number of nuclei in the sample were then checked using a Luna Automated Cell Counter. After adding 5 μl of Hoechst (1 mg/ml stock) per 1 ml of the sample and incubation for 30 min, Hoechst-positive particles in the sample were sorted using a FACS-ARIA II (BD) with a cooled chamber. After adjusting the volume of collected ~ 10 K Hoechst-positive events in 25.1 μl RT mix to 80 μl by adding 8.3 μl RT enzyme C and nuclease-free water, we followed the single-cell protocol provided by the manufacturer (10× Genomics), similar to scRNA-seq^23,24^.

### Single-cell and single-nucleus RNA sequencing and read processing

10× Chromium libraries were prepared according to the manufacturer’s protocol (10× Genomics), and sequencing libraries were sequenced on an Illumina HiSeq 2500 system according to the manufacturer’s instructions (Illumina). Both reads were aligned to the GRCh38 human genome reference sequence and quantified using Cellranger (version 3.1.0). Further analyses were conducted using Seurat software (version 3.1.4).

### Single-cell and single-nucleus RNA sequencing data analysis

Doublets were identified using Scrublet^25^ and filtered out with low-quality libraries that had fewer than 200 detected genes for tissue samples. Thereafter, cells from tissue samples with more than 20% mitochondrial transcripts were excluded from the analysis. Raw feature counts were then log-normalized, scaled, and subjected to linear dimensional reduction using principal component analysis. We applied “FindAnchor” and “Integrate” for colon tissues using PCs embedded into a space with reduced dimensionality to adjust for batch corrections across datasets. Dimensional reduction in a two-dimensional space was implemented using “RunUMAP.” We then identified clusters of cells using the “FindClusters” function of Seurat, which applies a shared nearest neighbor modularity optimization-based clustering algorithm. Different cell type clusters were identified by performing the “FindAllMarkers” function for each cluster and were annotated based on the expression of representative markers. Module scores of specific gene sets were estimated using “AddModuleScore”. Functional enrichment analysis was performed on differentially expressed genes (DEGs) between the two platforms according to Gene Ontology (GO) biological process (BP) terms using clusterProfiler version 4.2.2.

### Bulk RNA sequencing data processing and analysis

Total RNA sequencing libraries for the SMC dataset were constructed using the TruSeq Stranded Total RNA Library Preparation Kit with Ribo-Zero Gold (Illumina). Sequencing was performed in 100 bp paired-end mode on a HiSeq 4000 system (Illumina) at 120 million reads per sample. ERCC RNA Spike-In Mixes (Thermo Fisher Scientific) were included for quality assurance, and the sequences were aligned to the human reference genome (GRCh38.p10). RNA reads were aligned using STAR v.2.5.3.a and quantified as transcripts per million values using RSEM v.1.3.0^26^. To infer tumor purity from the bulk tissue samples, we used ESTIMATE^27^.

### Hematoxylin and eosin staining

Two primary colon tumor tissues were placed in 4% paraformaldehyde for 48 h and embedded in paraffin, followed by slicing into 5 μm sections. Tissue sections were deparaffinized and hydrated. The tissues were mounted on slides and stained with hematoxylin and eosin (H&E). The sections were rinsed and dehydrated using gradient alcohol and xylene. The stained sections were scanned under a high-resolution microscope attached to a slide-scanning platform (Aperio ScanScope XT).

## Results

### A comparison of two single-cell RNA sequencing platforms

We compared two single-cell RNA sequencing platforms, and the same samples from two types of surgically resected tissues were tested (four colon tissue samples and four liver tissue samples). The scRNA-seq experiment was conducted according to the conventional manufacturer’s protocol. In the case of the snRNA-seq experiment, we prepared pure nuclear samples to ensure that the nuclear suspensions were not aggregated, and to exclude debris and multiplets (Supplementary Fig. 1a), we used a combination of various nuclear isolation protocols for single-cell RNA-seq experiments, referred to as the ‘Frankenstein’ protocol by Luciano Martelotto^23^. This protocol was developed to prepare nuclei with a small sample size. Moreover, the preparation time was short, which may avoid the introduction of additional stress to fragile nuclei. After the nuclear suspension from the homogenized tissue was ready, Hoechst 33342 nuclear staining solution was added. Using flow cytometry, we gated on singlet and Hoechst+ nuclei and collected a specific target number of nuclei, calculated to reflect the nuclear recovery factor in a 96-well plate containing 10× RT buffer (Supplementary Fig. 1b).

Although each sample type was tested in two experiments, the libraries were sequenced and computationally analyzed together (Fig. 1a). On each platform, we collected 16,532 colon tissue cells for snRNA-seq and 12,526 colon tissue cells for scRNA-seq. We collected 13,144 liver tissue cells for snRNA-seq and 12,313 liver tissue cells for scRNA-seq. The average number of cells that passed QC and other sequencing parameters of cells that passed QC are reported in Supplementary Table 2.

Total transcript numbers were calculated and mapped to different aligned regions (exon, intergenic, and intron regions) using RNA-SeQC^28^. Most of the mapped reads were intronic under snRNA-seq, whereas most were exonic under scRNA-seq (Fig. 1b). The mitochondrial percentage under snRNA-seq was lower than that under scRNA-seq because mitochondria are cytosolic. These differences in mapped reads and mitochondrial percentages between the two platforms showed the same trends as previous results^13,21^.

### Profiling cell types of colon tissues and liver tissues

**Fig. 2:**
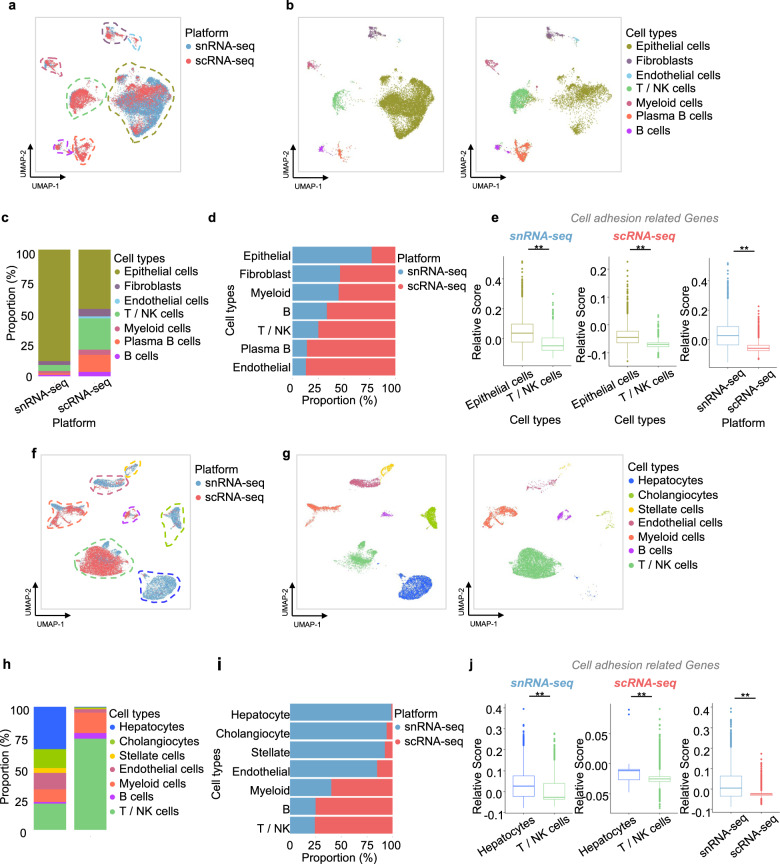
Diversity and proportion of cell types in the scRNA-seq and snRNA-seq results for colon tissues (a–e) and liver tissues (f–j). **a** UMAP embedding of scRNA-seq and snRNA-seq profiles of all colon tissues, colored according to cells or nuclei, **b** or the unsupervised clustering of snRNA-seq (left) and scRNA-seq (right). **c** Proportions of cells of different subsets for the two platforms. **d** Fractions of cells and nuclei in each cluster. **e** Module scores of cell adhesion-related genes for specific cell types under snRNA-seq (left) and scRNA-seq (middle) and for each platform (right) (**p ≤ 0.01; Wilcoxon signed-rank test). **f** UMAP embedding of scRNA-seq and snRNA-seq profiles of all colon tissues, colored according to cells or nuclei (**g**) or the unsupervised clustering of snRNA-seq (left) and scRNA-seq (right). **h** Proportions of cells of different subsets for the two platforms. **i** Fractions of cells and nuclei in each cluster. **j** Module scores of cell adhesion-related genes for specific cell types under snRNA-seq (left) and scRNA-seq (middle) and for each platform (right) (**p ≦ 0.01; Wilcoxon signed-rank test).

We performed an integrated analysis to identify differences in cell types and proportions across the different platforms. After filtering the cells, 21,226 colon tissue cells were analyzed (snRNA-seq, 13,580; scRNA-seq, 7646). After batch correction by using functions embedded in the Seurat packages, we defined seven major types of colon tissue cells using cell type-specific canonical marker genes previously defined in the literature^29^: epithelial cells, fibroblasts, endothelial cells, T/natural killer (NK) cells, myeloid cells, and B cells (Fig. 2a and Supplementary Fig. 2a). Projecting the results of the two platforms onto UMAPs revealed that the same cell types were conserved (Fig. 2b). However, the different platforms revealed distinct cell proportions (Fig. 2c, Supplementary Table 3). In particular, snRNA-seq showed much better sensitivity in the detection of epithelial cells, whereas scRNA-seq had much higher sensitivity in the detection of immune cells (Fig. 2d). The structures of epithelial cells are vulnerable to stress during dissociation as they are connected to each other. Therefore, stress-related genes, apoptosis genes, and genes related to heat shock proteins^21^ were detected sensitively by scRNA-seq, and module scores were calculated from the integrated expression values (Supplementary Fig. 3a). The module scores of cell adhesion-related genes^30^ for specific cell types were calculated to evaluate the adhesion of epithelial cells (Supplementary Table 4). Because molecules of the cadherin family are associated with cell‒cell adhesive bonds in solid tissues, the module score of epithelial cells was higher than that of T/NK cells on both platforms (Fig. 2e, left, middle). In addition to epithelial cells, fibroblasts and endothelial cells showed higher scores for adhesion-related genes in the snRNA-seq analysis (Supplementary Fig. 3b). Because scRNA-seq is limited by the destruction of the cell architecture, the module score of scRNA-seq was lower than that of snRNA-seq (Fig. 2e, right). To systemically validate this, we performed GO analysis of the associated biological pathways of DEGs identified between the two platforms as performed in previous studies^16,21^. Unsurprisingly, the upregulated genes identified by snRNA-seq were related to the regulation of cell-substrate adhesion and cell junction assembly. In contrast, the upregulated genes identified by scRNA-seq were related to responses to oxidative stress and the regulation of the apoptotic signaling pathway, potentially reflecting dissociation-related cell stress (Supplementary Fig. 3c).

To validate these findings in our colon tissues, we profiled another tissue type, metastatic liver tissues. Experiments and analyses using both platforms similar to those performed in colon tissues were conducted on four different metastatic liver tissues from three colon cancer patients. After filtering the cell types, 24,529 liver tissue cells were analyzed (snRNA-seq, 12,873; scRNA-seq, 11,656). We identified seven cell types using marker genes previously defined in the literature^31^: hepatocytes, cholangiocytes, stellates, and immune cells (Fig. 2f, Fig. 2g and Supplementary Fig. 2b). This shows that the two platforms revealed similar diversity (Fig. 2h) and different proportions (Supplementary Table 3). Because liver tissues are typically solid tissues, we hypothesized that they might be vulnerable to stress from dissociation. As expected, hepatocytes, cholangiocytes, and stellate cells were sensitive to snRNA-seq, whereas immune cells were sensitive to scRNA-seq (Fig. 2i). The module score of hepatocytes was higher than that of T/NK cells on both platforms (Fig. 2e, left and middle). In addition, cholangiocytes, stellate cells, and hepatocytes showed higher scores for adhesion-related genes in the snRNA-seq analysis than in the scRNA-seq analysis (Supplementary Fig. 3e). The experimental protocol for scRNA-seq resulted in cell stress (Supplementary Fig. 3d), validating the low module scores of the adhesion genes (Fig. 2j, right). We further validated the functional enrichment of the GO:BP terms of the DEGs between the two platforms in metastatic liver tissues. The dominant genes identified in snRNA-seq were related to cell–substrate adhesion and junction assembly as well as cell–matrix adhesion, where the last association indicated that snRNA-seq could achieve better coverage of adherent cell types than scRNA-seq (Supplementary Fig. 3f). This phenomenon was consistent with our primary findings.

### Clear transcriptional differences in epithelial cells of colon tissues

**Fig. 3:**
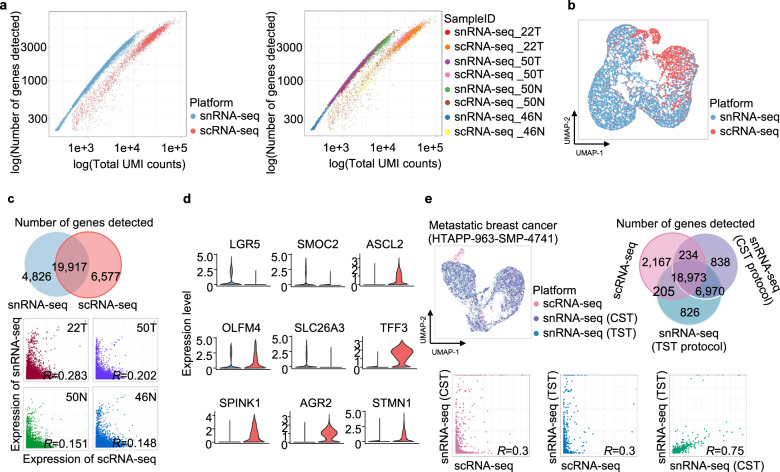
Transcriptional differences between the two platforms. **a** Total UMI counts of the number of detected genes plotted using different platforms (left) and different samples (right). **b** UMAP embedding of scRNA-seq and snRNA-seq profiles of epithelial cells extracted from colon tissues, color coded by platform. **c** Numbers of genes detected on common and distinct platforms (upper) and the low correlation of gene expression between two platforms across different samples (lower) (Pearson correlation). **d** Violin plot of the expression levels of various epithelial differentiation stage markers. **e** Independent external validation of breast cancer data for snRNA-seq and scRNA-seq in parallel. *CST* CHAPS with salts and Tris, *TST* tween with salts and tris.

The differences in cell proportions in solid tumors could be explained by the characteristics of their adhesive structure and apoptotic stress during the dissociation process. To obtain a deeper understanding of the molecular differences between the two platforms, we attempted to classify 14,910 epithelial cells (snRNA-sq, 11,983 cells; scRNA-seq, 2927 cells) of colon tissues based on their differentially expressed genes. The results showed varying transcriptional patterns. snRNA-seq revealed more genes with the same total unique molecular identifier (UMI) counts (Fig. 3a). The results of the two platforms were projected onto UMAPs after integrating all epithelial cells (Fig. 3b). When the analysis was limited to epithelial cells, only 20 and 25% of genes were identified by snRNA-seq and scRNA-seq, respectively. The expression of genes detected by both platforms was weakly correlated within all four sample types. This indicates that they showed different expression levels of the same gene (Fig. 3c). Interestingly, they showed differences in the enrichment of intestinal differentiation stage genes^29^. *LGR5* is an intestinal stem cell marker that was enriched in epithelial cells according to snRNA-seq, and *ASCL2*, another stem cell signature marker, was enriched according to scRNA-seq. *SLC26A3* is a marker of mature colonocytes enriched in epithelial cells that was enriched by snRNA-seq. *TFF3* is another differentiated colonocyte marker that was enriched by scRNA-seq (Fig. 3d). To validate our findings in an independent external study, we investigated metastatic breast cancer (MBC) originating from epithelial cells and processed the cells simultaneously for snRNA-seq and scRNA-seq^13^ (Sample ID: HTAPP-963-SMP-4741). Following the analysis conducted as discussed previously, we evaluated 11,037 epithelial cells (snRNA-sq [CST protocol], 6964 cells; snRNA-sq [TST protocol], 3468 cells; scRNA-seq, 605 cells) in breast cancer tissues. The results of snRNA-seq obtained by the two protocols shared a greater fraction of genes with each other than with the scRNA-seq results. In addition, the expression levels of genes detected by the three platforms showed a biased correlation between snRNA-seq and scRNA-seq. While the levels recorded in the scRNA-seq analysis were weakly correlated with the results of snRNA-seq under both protocols, the results of the two snRNA-seq protocols showed a higher correlation with each other (Fig. 3e). This indicates that the findings in our primary cohort could be reproduced in other cancer types. Taken together, discrepancies derived from the detailed subclassification of specific cells indicate that the decision to use a certain platform is crucial for achieving the research goal. snRNA-seq not only recovered a greater number of epithelial cells but also showed more intensive gene expression than scRNA-seq.

### Comparison of methods for profiling cell types

**Fig. 4:**
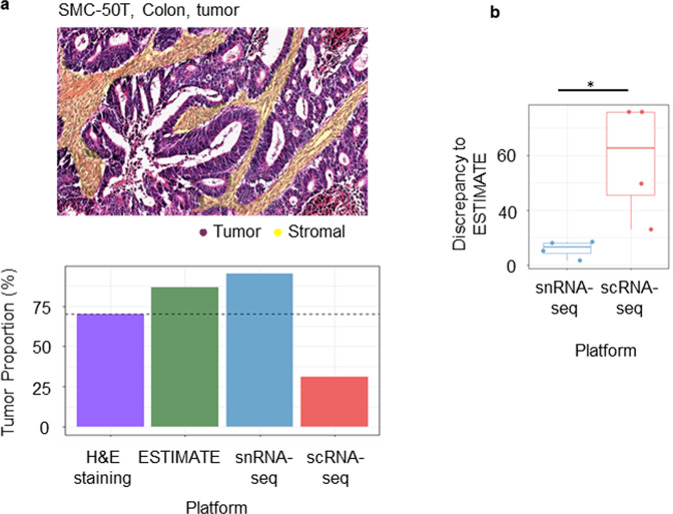
Comparison of bulk tissues with the sequencing results of each platform. **a** Annotated colon tumor tissue (SMC-50T), hematoxylin and eosin (H&E, ×100) staining. The annotations indicated a region of stromal cells (yellow) and tumor cells (remaining cells) (upper), and the percentage of tumor cells (epithelial cells) estimated by H&E staining, bulk-RNA sequencing (ESTIMATE), snRNA-seq, and scRNA-seq (lower). **b** Differences in the tumor percentages between each platform and the bulk sequencing results of colon tissues (*n* = 4) (*, 0.01 < *p* ≤ 0.05; Wilcoxon signed-rank test).

Pathologists routinely determine the cellularity of clinical tumor samples through the visual evaluation of hematoxylin- and eosin-stained slides (Fig. 4a, upper). Before the development of scRNA-seq, the estimation of RNA expression levels to decipher stromal, immune and tumor proportions, referred to as “purity”, was conducted based on bulk RNA-seq data^27^. We identified the cell types and inferred the epithelial proportion from the colon tumor tissues (SMC-50T, representative sample of two primary colon tumor tissues) using four distinct platforms. We observed that the scRNA-seq results were the most inconsistent among all platforms concerning the estimation of tumor proportion (Fig. 4a, under). In addition, the four colon tissues showed epithelial cell proportions similar to those identified by snRNA-seq. In contrast, scRNA-seq values showed a large discrepancy with the values inferred by ESTIMATE, which interprets tumor purity based on bulk RNA-seq values (Fig. 4b). These results indicate that snRNA-Seq better reflects bulk RNA-seq and pathological results in terms of tumor purity, which may be explained by the high module score of snRNA-Seq, as described earlier.

## Discussion

In this study, we compared scRNA-seq and snRNA-seq using two tissue types. We selected these tissues because they consist of various cell types that must be identified and dissociated in problematic tissues (Fig. 1). The obtained results are more effective for deciphering complex biological systems than previously reported data from cell lines, human peripheral blood mononuclear cells, and mouse cortex nuclei^32^.

Although our work agrees with a previous study demonstrating that the two platforms are consistent in identifying cell types, we revealed conditional advantages of one platform over the other in different scenarios. First, because the platforms show different cell distributions, certain cell classifications could be oriented toward one platform (Fig. 2). For example, Wu et al. reported the advantages of snRNA-seq over scRNA-seq and illustrated novel cell states and a rare cell type in an inflamed fibrotic kidney^21^. Through snRNA-seq, we also captured rare cell types, such as cholangiocytes and hepatic stellate cells, in liver tissues. These cells were reported to constitute less than 1% of the scRNA-seq map^31^. Second, we observed different lineage-specific expression levels of colonic epithelial cell differentiation marker genes between the two platforms (Fig. 3). For example, Nicola et al. showed that snRNA-seq is not suitable for detecting microglial activation genes in humans^22^. Although endothelial cells are highly adherent, their proportion was higher in the scRNA-seq results than in the snRNA-seq results for colon samples. Endothelial cells were effectively recovered by snRNA-seq limited to liver tissues containing many endothelial cells, due to their anatomy and functions. Taken together, the results indicate that decision-making in deploying these tools depends on “target” cell types or genes. This study will allow future studies to reduce time wastage in optimal methodology testing. When interpreting the differences in the expression levels of epithelial lineage markers, it remains unclear which platform is able to classify them at more detailed levels, such as among goblet cells, colonocytes, tuft cells, and enteroendocrine cells. In addition to the genes related to epithelial cell lineages identified in this study, other differentially expressed genes and their functions need to be explored. This may explain why scRNA-seq showed high UMI counts, except for the transcripts encoded in the mitochondrial genome. Third, we simultaneously applied four methodologies to identify the cell-type constitution of a single patient (Fig. 4). It is meaningful to compare single-cell transcriptional approaches with two platforms that have been prevalent in clinical studies on diagnosing or treating patients. As a result, the results for epithelial tumor cells obtained by scRNA-seq were not consistent with pathology and bulk RNA-seq results, suggesting that scRNA-seq may overestimate the proportion and functions of immune cells in the tumor microenvironment.

In conclusion, we identified the characteristics and differences of the cell type distributions generated by scRNA-seq and snRNA-seq. For matched tissues, the two platforms showed similar diversity but different proportions of cell types. This could be explained by the disruption of cytoplasmic contents during scRNA-seq, which resulted in differences in the expression scores of adhesion genes. The enrichment of epithelial cells in the colon by snRNA-seq explains the discrepancy in the differentiation of epithelial cells in the colon. This enrichment was also well matched with the images of hematoxylin and eosin staining and the estimated distribution of cell types in bulk tissue. Our results reveal the reasons for the characteristics and differences of the cell type distributions obtained with each platform and their specific applications. They also imply that it is necessary to select a specific platform according to the research purpose.

## Supplementary information

Supplementary material

## Data Availability

Due to the regulations of the institution, individual-level sequencing and detailed clinical data from this research cannot be uploaded to the public repository domain. It will be shared upon request to researchers who provide an approved proposal. Requests for access to the data can be made to woongyang.park@samsung.com. Requestors will be provided with assistance on how data can be accessed following the submission of the research proposal.
