# Supplementary material for: Comparison of cell type distribution between single-cell and single-nucleus RNA sequencing: enrichment of adherent cell types in single-nucleus RNA sequencing
Source: Exp Mol Med. 2022 Dec 2;54(12):2128–34. doi: 10.1038/s12276-022-00892-z (PMC9794763; doi:10.1038/s12276-022-00892-z)
Supplement: Supplementary file 1 — Supplementary material [file 12276_2022_892_MOESM1_ESM.pdf]

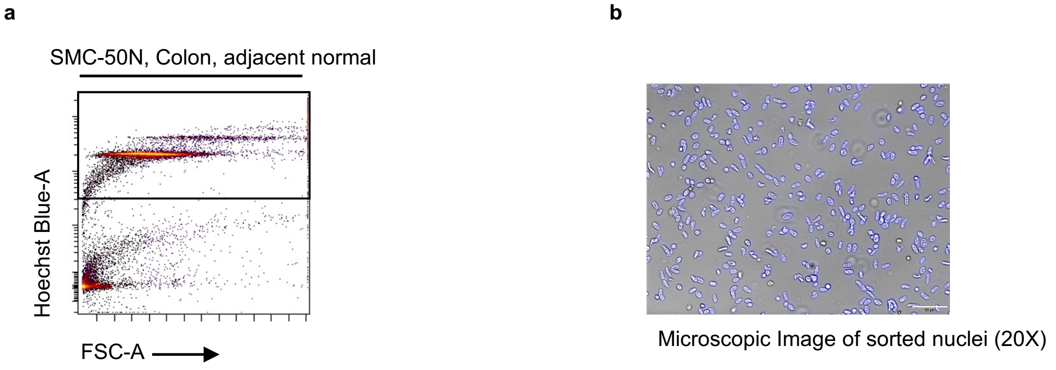

Figure S1. Nuclei sorting. (a) Hoechst blue-A gating of nuclei to deplete cell debris. (b) Microscopic image of sorted nuclei (20X).

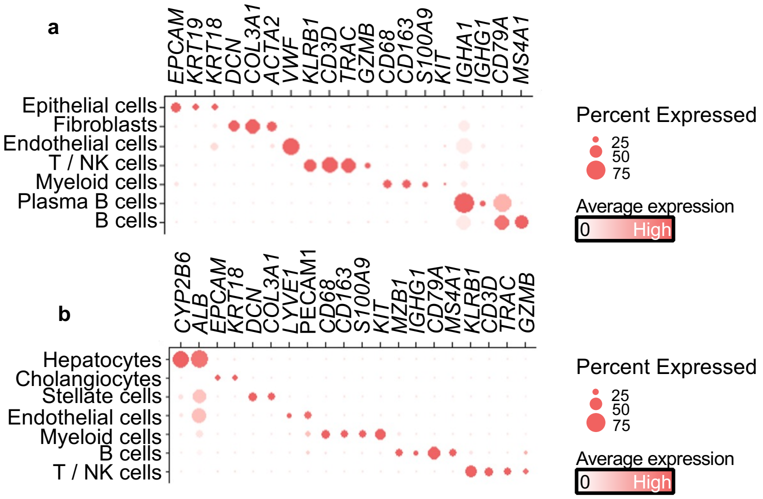

Figure S2. Identification of the cell types. Average expression of canonical marker genes for colon tissues (a) and liver tissues (b).

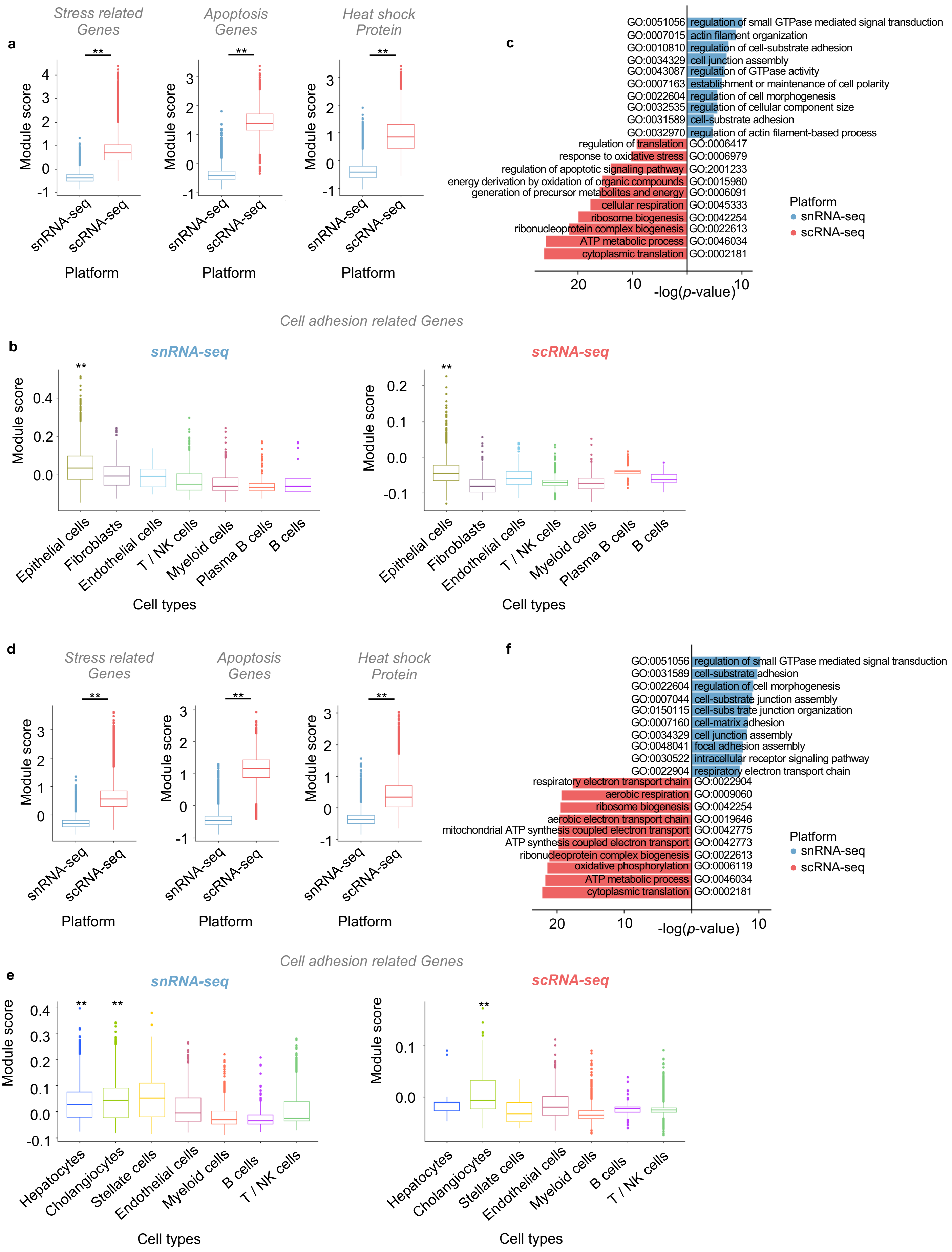

Figure S3. Enriched genes and the module score of cell adhesion related genes for all cell types clustered in snRNA-seq (left), scRNA-seq (right) for colon tissue (a,b) and liver tissue (d,e). Colon epithelial cells shows statistically significant difference of scores with all immune cell types on both platforms. Cholangiocytes shows statistically significant difference of scores with all immune cell types on both platforms (**, $p \leq 0.01$; Wilcoxon signed-rank test). Gene Ontology (GO) analysis on differentially expressed genes (DEGs) associated Biological Process (BP) between two platforms for colon tissue (c) and liver tissue (f).

Table S1. Patient information in the 5 SMC datasets.

Patient	Tumor Colon	Normal Colon	Tumor Liver	Normal Liver	Diagnosis	Metastasis	Gender	Age	TNM stage	Stage	Anatomic region	MSI	Pathological subtype
SMC22	O (SMC-22T)	-	-	-	Colorectal cancer	-	M	76	T3 N1b M0	IIIB	sigmoid	MSS	Adenocarcinoma, moderately differentiated
SMC50	O (SMC-50T)	O (SMC-50N)	-	-	Colorectal cancer	-	F	74	T3 N0 M0	IIA	ascending	MSS	Adenocarcinoma, well differentiated
SMC37	-	-	O (SMC-37LMT)	O (SMC-37LMN)	Colorectal cancer	O	M	23	T3 N2b M1a	IVA	ascending	MSI-H	Adenocarcinoma, moderately differentiated
SMC46	-	O (SMC-46N)	-	O (SMC-46LMN)	Colorectal cancer	O	M	70	T3 N1b M1a	IVA	sigmoid	MSS	Adenocarcinoma, moderately differentiated
SMC99	-	-	-	O (SMC-99LMN)	Colorectal cancer	O	F	57	T4a N2a M0	IIIB	sigmoid	-	Adenocarcinoma, moderately differentiated

Table S2. Quality Overview.

Tissue	Platform	Average cell No. (sd)	Average Reads (sd)	Average nFeature_RNA (sd)	Average nCount_RNA (sd)	Average Doubletscore (sd)
Colon (n=4)	snRNA-seq	4,133	118,162,310	1,301.037	2,166.683	0.098
		(1,526.146)	(7,458,245)	(895.284)	(2,093.413)	(0.056)
	scRNA-seq	3,131.5	28,298,754	1,444.640	9,036.805	0.058
		(1,148.43)	(19,639,348)	(1,463.436)	(10,761.790)	(0.052)
Liver (n=4)	snRNA-seq	3,286	2,980,270	1,378.967	2,690.7	0.072
		(1,035.849)	(3,507,855)	(859.888)	(2,835.492)	(0.070)
	scRNA-seq	3,078.250	97,476.5	1,144.327	3,932.441	0.067
		(1,718.872)	(115,176)	(589.765)	(3,647.015)	(0.061)

Table S3. Cell fraction.

Tissue	Celltype	Platform	Percent
Colon	Epithelial	sNuc-seq	88.240
	Fibroblast	sNuc-seq	2.850
	Endothelial	sNuc-seq	0.133
	T_NK	sNuc-seq	4.691
	Myeloid	sNuc-seq	1.885
	PlasmaB	sNuc-seq	1.230
	B	sNuc-seq	0.972
	Epithelial	sCell-seq	38.281
	Fibroblast	sCell-seq	5.885
	Endothelial	sCell-seq	1.569
	T_NK	sCell-seq	32.828
	Myeloid	sCell-seq	4.094
	PlasmaB	sCell-seq	13.929
	B	sCell-seq	3.414
Liver	Hepatocyte	sNuc-seq	34.297
	Cholangiocyte	sNuc-seq	15.435
	Stellate	sNuc-seq	3.822
	Endothelial	sNuc-seq	13.361
	Myeloid	sNuc-seq	10.231
	B	sNuc-seq	1.352
	T_NK	sNuc-seq	21.502
	Hepatocyte	sCell-seq	0.498
	Cholangiocyte	sCell-seq	1.055
	Stellate	sCell-seq	0.343
	Endothelial	sCell-seq	2.660
	Myeloid	sCell-seq	16.730
	B	sCell-seq	4.470
	T_NK	sCell-seq	74.245

Table S4. Gene lists for estimating module score.

Genes	Contents
Stress related Genes	*GADD45B, EGR1, NR4A1, ATF3, FOS, FOSB, JUN, JUNB, JUND*
Apoptosis Genes	*CRIP1, PPP1R15A , ATF3, DYNLL1, LER3, LMNA , UBB , UBC , JUN, JUNB, JUND, TPT1*
Heat shock Proteins	*DNAJA1, HSP90AB1, HSP90B1, HSPA5, HSPA8, HSPB1 , HSPA1A, HSPA1B*
Cell adhesion related Genes	*CDH1, CDH10, CDH11, CDH13, CDH15, CDH16, CDH17, CDH2, CDH24, CDH3, CDH4, CDH6, CDH7, CDH8, CDH9, CDHR2, CDHR5*
